# Effects of Lysolecithin on Growth Performance, Nutrient Digestibility, Serum Biochemical Indices, and Rumen Environment in Holstein Calves

**DOI:** 10.1002/fsn3.71320

**Published:** 2025-12-08

**Authors:** Zhigao An, Shijia Pan, Bo He, Junhong Wang, Yanming Wang, Shijie Sun, Chong Wang

**Affiliations:** ^1^ College of Animal Science and Technology & College of Veterinary Medicine, Zhejiang A&F University, Key Laboratory of Applied Technology on Green‐Eco‐Healthy Animal Husbandry of Zhejiang Province Zhejiang Hangzhou China; ^2^ Yueqing Agriculture and Forestry Technology Service Center Yueqing China; ^3^ College of Animal Sciences Zhejiang University Zhejiang Hangzhou China; ^4^ Kemin (China) Technologies Co. Ltd. Zhuhai China

**Keywords:** growth performance, Holstein calves, lysolecithin, nutrient digestibility, rumen environment

## Abstract

This study was to evaluate the effects of lysolecithin on the growth performance, serum biochemical indices, and rumen environment of Holstein calves. A total of 26 calves were divided into a control group with a basal diet (CON) and a treatment group that added 5 g/d lysolecithin (LY). Compared to the CON, the proportion of feces in the middle sieve was higher, and the proportion of the bottom sieve was lower in the LY (*p* < 0.05). Serum biochemical parameters indicated that the LY had higher albumin, total antioxidant capacity, and total cholesterol, whereas non‐esterified fatty acid, blood urea nitrogen, and triglyceride were lower than the CON (*p* < 0.05). The NH_3_‐N of rumen fluid in the LY was lower than that of the CON (*p* < 0.05). The rumen fluid percentage of butyrate, iso‐butyrate, valerate, and iso‐valerate in the LY was higher than in the CON (*p* < 0.05), with a tendency for lower propionate compared to the CON (*p* < 0.10). In the rumen microorganism, the abundance of *Ruminococcu*s in the LY was higher than in the CON (*p* < 0.05). Mantel test indicated that the percentage of acetate was found significantly positively correlated with *Candidatus_Saccharimonas*, whereas propionate exhibited positive correlations with *Ruminococcus_gauvreauii_group*, *Candidatus_Saccharimonas*, and *unclassified Lachnospiraceae* (*p* < 0.05). Additionally, the *NK4A214_group* showed a significant positive correlation with the acetate/propionate, and the concentration of iso‐valerate was positively correlated with *norank_f__norank_o__Clostridia_UCG‐014* (*p* < 0.05). In summary, dietary lysolecithin supplementation could positively influence the rumen microbial community, enhance rumen fermentation, and improve antioxidant ability, without affecting growth performance in Holstein calves.

## Graphical abstract

Lysolecithin increased serum albumin, total antioxidant capacity, and total cholesterol, while decreased non‐esterified fatty acid, blood urea nitrogen and triglyceride. Lysolecithin increased percentage of butyrate, iso‐butyrate, valerate and iso‐valerate in rumen fluid. The abundance of *Ruminococcus* in the addition of lysolecithin was higher compared with CON.

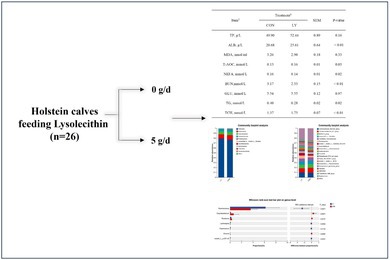

## 1 Introduction

The health and growth of dairy calves are paramount in rearing programs and are influenced by numerous factors, with nutrition being a critical component of their health and growth. The growth performance of dairy calves has a direct effect on their future production performance (Kertz et al. 2017). Of all the nutrients, lipids are an important source of energy for animals, and provide a large amount of energy. Compared to adult animals, juvenile animals have a lower capacity for lipid absorption (Asokapandian et al. 2021). The supplementation of emulsifiers in the diet can enhance lipid digestibility and promote growth (Wang et al. 2024). Emulsifiers, as feed additives, can enhance fat digestion, thereby improving feed energy utilization and promoting rapid growth (Gholami et al. 2024).

Lysolecithin belongs to lecithin with excellent emulsifying properties. It is effective that lysolecithin can facilitate the formation of mixed micelles containing water‐soluble nutrients in the gastrointestinal tract and improve the absorption of nutrients (Weng et al. 2022). Currently, the use of lysolecithin as a feed additive is primarily concentrated on monogastric animals. Previous studies have demonstrated that lysolecithin has enhanced nutrient digestibility and growth performance of broilers (Zhang, Zhang, et al. 2022). Moreover, lysolecithin can protect liver health, leading to enhanced antioxidant capacity and immunity in broilers (Cai et al. 2024). In weaned piglets, lysolecithin may improve growth performance by reducing lipid breakdown and alleviating oxidative stress (Liu et al. 2023). Supplementing diets with lysolecithin can modify the fatty acid composition in milk, helping to maintain a balanced gut microbiota in piglets and enhancing their survival rates (Jang et al. 2020). Similarly, dietary lysolecithin can enhance growth performance, improve liver lipid metabolism, and modulate inflammatory responses in juvenile large yellow croaker (Weng et al. 2022). For ruminants, previous studies have indicated that lysolecithin can increase the abundance of *Ruminococcus* and butyrate in beef cattle feces, which may account for the observed increase in average daily gain (Zhang et al. 2022a). Additionally, the incorporation of lysolecithin into the diet can improve nutrient digestibility, leading to enhanced growth and improved feed efficiency, while enhancing antioxidant capacity (Zhang et al. 2022b). Previous studies have reported that lysolecithin can improve growth performance while also preventing diarrhea in dairy calves (Reis et al. 2021).

Although lysolecithin application has been extensively studied in monogastric animals, research on its effects in ruminants remains limited. Given lysolecithin's emulsifying role and its positive effects in other animals, we hypothesized that dietary lysolecithin supplementation would change growth performance, nutrient digestibility, rumen fermentation parameters, and microbial community composition in dairy calves.

## 2 Materials and Methods

### 2.1 Experimental Animals and Design

The experimental procedure was approved by the animal care protocol was approved by the Animal Ethics Committee of Zhejiang A&F University (ZAFUAC202451), and humane animal care and handling procedures were implemented in the experiment. Twenty‐six healthy male Holstein calves (body weight = 213.53 ± 69.56) from JiaXing Rongzhong dairy farm, China. They were randomly allotted to two treatments: (1) basal diet (CON), (2) basal diet added 5 g/d lysolecithin (LY) (Lysoforte, Kemin (China) Technologies Co. Ltd. 30% lysolecithin). Following a 7‐day acclimation period with a basal diet, the 9‐week experimental phase commenced. During the study, total mixed ration (TMR) was offered 3 times daily (07:00, 12:00, and 18:00), adjusted to ensure approximately 10% refusal. The TMR compositions are shown in Table 1. Calves had free access to water and diets throughout the entire experiment.

**TABLE 1:** Composition of trial diets.

Item	Content, %
Ingredient (% of DM)	
Corn silage	39.94
Corn grain	18.83
Alfalfa hay	15.21
Oat hay	7.98
Wheat bran	4.50
Soybean meal	4.32
Cottonseed meal	3.20
Rapeseed meal	3.20
DDGS	1.41
Premix^a^	1.41
Nutrient compositions (% of DM)	
CP	10.14
EE	2.14
ADF	13.52
NDF	21.63
Ash	3.34

^a^Co, 20–30; Cu, 1600–2400; I, 55–80; Fe, 2400–6000; Mn, 2560–3840; Se, 50–80; Zn, 32,000–4800 mg/kg; vitamin A, 580,000–860,000 kIU/kg; vitamin D, 220,000–340,000 IU/kg; and vitamin E 5760–8640 IU/kg.

### 2.2 Sample Collection

Blood and rumen fluid were collected on the trial termination day. Two hours after morning feeding, blood samples were collected from all calves through the jugular vein in 10 mL vacuum tubes containing heparin sodium anticoagulant. Serum samples were collected by centrifugation at 3000 *g* for 15 min at 4°C and stored at −80°C until later determination of serum indexes (Roshanzamir et al. 2020). Rumen fluid was collected before morning feeding using an oral gastric tube, discarding the first 200 mL to avoid saliva contamination, and filtered through 4 layers of gauze. After that, the samples were stored at −80°C for the determination of rumen fermentation parameters and microorganisms.

### 2.3 Feed Chemical Analyses

The feed samples were dried in a 65°C oven for 48 h, then vacuum‐sealed in plastic containers and stored at 4°C. Before analysis, samples were ground through a 1 mm sieve. The subsequent detection methods can be referred to in the previous reports (Pan et al. 2025).

### 2.4 Growth Performance Measurement

Dry matter intake (DMI) was measured by subtracting the quantity of rations that were refused from the number of rations initially offered, and then dividing the resulting value by the total number of calves. The body weight, chest girth, and body diagonal were measured at weeks 4, 7, and 10. Average daily gain (ADG) was calculated by dividing the weight gained by the number of feeding days. The chest circumference was measured using a tape measure around the widest part of the ribcage, situated just behind the front legs, and the oblique length refers to the distance from the shoulder tip to the hip tip (Peng et al. 2024).

### 2.5 Serum Biochemical Indices

Biochemical index includes total protein (TP), albumin (ALB), blood urea nitrogen (BUN), malondialdehyde (MDA), total antioxidant capacity (T‐AOC), glucose (GLU), nonesterified fatty acid (NEFA), triglyceride (TG), total cholesterol (TCH), and alkaline phosphatase (ALP) using commercial kits in accordance with manufacturer instructions (Jiancheng Bioengineering Institute, Nanjing, China). Globulin (GLB) was calculated by subtracting the ALB from the TP.

### 2.6 Analysis of the Fecal Sample

During the experiment, the fecal scoring scale was recorded weekly according to a standard scoring procedure (4 = normal feces; 3 = semi‐formed feces; 2 = loose feces; and 1 = watery feces) (Xin et al. 2021). In addition, 3 days ahead of the experiment's completion, the fresh feces of 12 Holstein calves in each group were collected from the rectum at the same time each day, washed with water, and passed through three layers of feces sieve in turn. Among them, the three‐layer sieve assembly constitutes the core component, with mesh apertures of the upper, middle, and lower tiers measuring 4.76, 2.38, and 1.59 mm, respectively. The feces in the three‐layer sieve are weighed, and the relative proportions of each layer are calculated (Kljak et al. 2019).

### 2.7 Measurement of Rumen Fermentation

The volatile fatty acid (VFA) content in the rumen was determined by gas chromatography (Agilent 7890B, CA, USA) by adding 1 mL of 25% metaphosphoric acid to 5 mL of rumen fluid samples and mixing properly (Mao et al. 2023). The ammonia‐N concentration of rumen liquid was determined by the phenol blue method with an ultraviolet spectrophotometer (UV1901; Wavelength: 550 nm) (Novamsky et al. 1974).

### 2.8 Rumen Microorganisms

Total microbial genomic DNA was extracted from rumen fluid samples using a soil DNA kit (Omega Bio‐tek, Norcross, GA, U.S.), and the quality of the extracted DNA was evaluated by agarose gel electrophoresis. The bacterial 16S rRNA gene's V3–V4 hypervariable regions were amplified via PCR with the primers 338F (5′‐ACTCCTACGGGAGGCAGCA‐3′) and 806R (5′‐GGACTACHVGGGTWTCTAAT‐3′). The PCR products were separated on a 2% agarose gel, purified with the AxyPrep DNA Gel Extraction Kit (Axygen Biosciences, Union City, CA, USA), and quantified. Purified amplicons were subjected to sequencing on the Illumina MiSeq platform (Illumina, San Diego, USA). Raw sequencing reads were processed through quality filtering using FastQ and assembled with FLASH. Subsequent steps, including demultiplexing, quality control, and taxonomic labeling, were performed within the Qiime2 pipeline (version 2022.2). The 16S rRNA sequences were further filtered, and high‐quality reads were denoised into amplicon sequence variants (ASVs) using the DADA2 plugin in Qiime2 under recommended settings. Taxonomic classification of ASVs was conducted using a Naïve Bayes classifier integrated within Qiime2, with reference to the 16S rRNA database. To evaluate β‐diversity across ruminal microbial communities, principal coordinate analysis (PCoA) was applied on the basis of Bray–Curtis distances. Microbial composition differences between groups were assessed using linear discriminant analysis effect size (LEfSe).

### 2.9 Statistical Analysis

These data were analyzed by using SPSS 25.0 (SPSS Inc., USA). Correlations between rumen fermentation indicators and rumen microorganisms were analyzed using the Mantel test, and *p*‐value (Spearman's rank correlation coefficient) < 0.05 was considered to be significantly correlated. The growth performance was analyzed through repeated measures analysis. Fecal scores and the proportion of feces were analyzed through the Wilcoxon rank‐sum test. Serum biochemical index and rumen fermentation parameters were analyzed through one‐way ANOVA. The correlation between ruminal fermentation and rumen microorganisms was analyzed by the R “dplyr, linkET, ggplot2” software package. *p* < 0.05 was considered statistically significant, and trend was defined as 0.05 ≤ *p* < 0.10.

## 3 Results

### 3.1 Effects of Lysolecithin on the Growth Performance in Holstein Calves

The differences in growth performance and body measurements are shown in Table 2. The DMI and BW were not different between the two groups (*p* > 0.05). No significant differences in body measurements, including chest girth and body diagonal between the CON and lysolecithin (*p* > 0.05, Table 3).

**TABLE 2:** Effect of lysolecithin on feed intake of Holstein calves.

Item	Treatment^a^		SEM	*p*
	CON	LY		
DMI (kg/d)				
4th week	5.98	6.20	0.63	0.73
5th week	8.31	8.44	0.85	0.88
6th week	6.49	6.66	0.60	0.78
7th week	7.98	8.09	0.67	0.88
8th week	7.19	7.22	0.51	0.96
9th week	7.39	7.43	0.46	0.93
Average weekly DMI (4th–9th week)	7.22	7.34	0.27	0.84
BW (kg)				
1st week	205.49	221.57	13.19	0.57
4th week	235.41	255.30	14.13	0.49
7th week	269.15	286.85	14.16	0.54
9th Week	289.82	308.59	15.21	0.55
BW gain (1st–9th week)	84.33	87.02	3.78	0.73
Average daily gain (1st‐9th week)	1.34	1.38	0.06	0.73

Abbreviations: BW, body weight; DMI, dry matter intake.

^a^ CON, basal diet; LY, basal diet added 5 g/d lysolecithin.

**TABLE 3:** Effect of lysolecithin on body size parameters of Holstein calves.

Item	Treatment^a^		SEM	*p*
	CON	LY		
4th Week				
Chest girth, m	1.43	1.47	0.03	0.52
Body diagonal, m	1.15	1.17	0.02	0.56
7th Week				
Chest girth, m	1.49	1.54	0.03	0.42
Body diagonal, m	1.20	1.20	0.02	0.96
9th Week				
Chest girth, m	1.53	1.58	0.03	0.48
Body diagonal, m	1.23	1.24	0.01	0.87
Total gain (4th–9th weeks)				
Chest girth, m	0.10	0.11	0.00	0.55
Body diagonal, m	0.08	0.07	0.01	0.23

^a^ CON, basal diet; LY, basal diet added 5 g/d lysolecithin.

### 3.2 Effects of Lysolecithin on the Nutrient Digestibility in Holstein Calves

The results are presented in Table 4, the proportion of the top sieve for fecal in the LY was higher, whereas the proportion in the LY of the bottom sieve was lower than the CON (*p* < 0.05). In addition, there was no significant difference in the proportion of the middle sieve between groups (*p* > 0.05). No differences were observed for fecal scores by the treatments (*p* > 0.05).

**TABLE 4:** Effect of lysolecithin on feces score of Holstein calves.

Item	Treatment^a^		SEM	*p*
	CON	LY		
Fecal score				
4th week	2.92	3.08	0.08	0.54
5th week	3.23	3.00	0.12	0.45
6th week	3.15	3.08	0.10	0.84
7th week	3.08	2.85	0.09	0.36
8th week	3.08	2.92	0.08	0.54
9th week	3.08	3.15	0.10	0.76
Proportion, %				
Top sieve	7.31	8.70	0.43	0.11
Middle sieve	16.03	22.93	1.38	< 0.01
Bottom sieve	76.68	67.88	1.65	< 0.01

^a^ CON, basal diet; LY, basal diet added 5 g/d lysolecithin.

### 3.3 Effects of Lysolecithin on the Serum Biochemistry in Holstein Calves

The effects of lysolecithin on plasma biochemical indices are presented in Table 5. Compared with the CON, the ALB, T‐AOC, and TCH in LY were higher, whereas NEFA, BUN, and TG in the LY were lower (*p* < 0.05). However, no differences were observed in TP, MDA, and GLU by the treatments (*p* > 0.05).

**TABLE 5:** Effect of lysolecithin on plasma biochemical indices of Holstein calves.

Item	Treatment^a^		SEM	*p*
	CON	LY		
TP, g/L	49.90	52.44	0.89	0.16
ALB, g/L	20.68	25.61	0.64	< 0.01
MDA, nmol/ml	3.26	2.90	0.18	0.33
T‐AOC, mmol/L	0.13	0.16	0.01	0.03
NEFA, mmol/L	0.16	0.14	0.01	0.02
BUN, mmol/L	3.17	2.33	0.15	< 0.01
GLU, mmol/L	5.54	5.55	0.12	0.97
TG, mmol/L	0.40	0.28	0.02	0.02
TCH, mmol/L	1.37	1.75	0.07	< 0.01

Abbreviations: ALB, albumin; BUN, blood urea nitrogen; GLU, glucose; MDA, malondialdehyde; NEFA, nonesterified fatty acid; T‐AOC, total antioxidant capacity; TCH, total cholesterolTG, triglyceride; TP, total protein.

^a^ CON, basal diet; LY, basal diet added 5 g/d lysolecithin.

### 3.4 Effects of Lysolecithin on the Ruminal Fermentation in Holstein Calves

As reported in Table 6, the percentage of butyrate, iso‐butyrate, valerate, and iso‐valerate in the LY was higher than in the CON (*p* < 0.05). The NH_3_‐N of the LY was lower than that of the CON (*p* < 0.05). Propionate tended to decrease with the lysolecithin addition (*p* < 0.10).

**TABLE 6:** Effects of lysolecithin on rumen fermentation parameters of Holstein calves.

Item	Treatment^a^		SEM	*p*
	CON	LY		
NH_3_‐N, g/L	0.38	0.29	0.04	0.03
Total VFA, mmol/L	159.69	191.12	14.71	0.31
Acetate, %	52.45	51.83	1.47	0.86
Propionate, %	27.76	23.76	1.00	0.09
Butyrate, %	14.35	18.53	0.99	0.02
Iso‐butyrate, %	1.12	1.37	0.06	0.03
Valerate, %	2.03	2.61	0.12	0.01
Iso‐valerate, %	2.28	3.03	0.19	0.04
Acetate/Propionate	1.94	2.13	0.10	0.45

Abbreviation: VFA, Volatile fatty acids.

^a^ CON: basal diet; LY = basal diet added 5 g/d lysolecithin.

### 3.5 Effects of Lysolecithin on the Ruminal Microbiota in Holstein Calves

**FIGURE 1:**
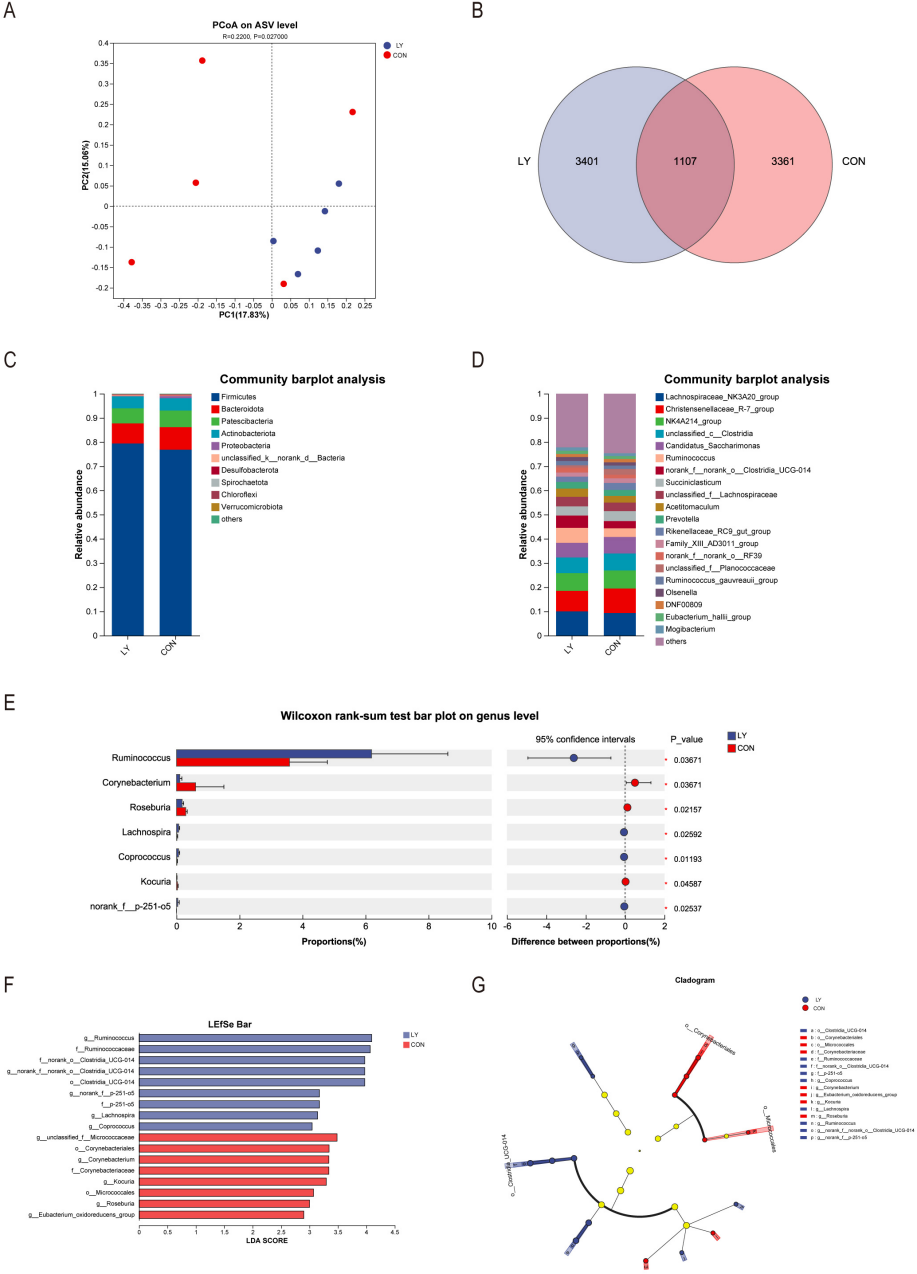
Effect of lysolecithin on ruminal microbiota in Holstein calves. CON, Basal diet; LY, basal diet added 5 g/d lysolecithin. (A) Plots of principal coordinates analysis (PCoA) comparing the overall rumen microbiota among two groups. (B) The Venn diagram shows the shared and unique bacterial ASVs in rumen samples in CON and LY. (C) Relative abundance of microbiota at the phylum level. (D) Relative abundance of microbiota at the genus level. (E) Wilcoxon rank‐sum test bar plot on genus level. (F) Cladogram showed the significantly different bacteria from the phylum to genus level. The nodes with different colors represent the microbes that are significantly enriched in the corresponding groups and have a significant influence on the difference among three groups. (G) Linear discriminant analysis (LDA) bar showed the impact of the abundance of each species on the difference among two groups. *p* < 0.05 and LDA > 2 were defined as significant differences.

No differences in the Ace, Chao 1, and Shannon index were observed between the CON and LY (*p* > 0.05, Table 7). The Simpson index tended to decrease in the LY (*p* < 0.10). The rumen microbiota composition is illustrated in Figure 1. PCoA indicated that no difference in β‐diversity by treatment (Figure 1A, *p* > 0.05). The Venn diagram results showed that 1107 ASVs were shared, and 3401, 3361 ASVs were unique to the LY and CON (Figure 1B), respectively. The ruminal dominant bacteria including *Firmicutes*, *Bacteroidota*, *Patescibacteria*, and *Actinobacteriota*, otherwise, no differences were noted between the CON and LY at the phylum level (Figure 1C, *p* > 0.05). At the genus level (Figure 1D,E), there were ruminal dominant genera in two groups, including *Firmicutes*, *Lachnospiraceae_NK3A20_group*, *Christensenellaceae_R‐7_group*, *NK4A214_group*, *unclassified_c__Clostridia*, *Candidatus_Saccharimonas*, and *Ruminococcus* (Figure 1D). Additionally, the relative abundances of *Ruminococcus*, *Lachnospira*, *Coprococcus*, and *norank_f__p‐251‐o5* in LY were higher, whereas the *Corynebacterium*, *Roseburia*, *Kocuria*, and *unclassified_f__Micrococcaceae* in the LY were lower compared with the CON (Figure 1E, *p* < 0.05). It was shown that nine clades in the LY, whereas eight clades in the CON were influenced by the structure of the rumen bacterial community (Figure 1F,G). The microbiota in the LY were *g__Ruminococcus, f__Ruminococcaceae, g__norank_f__norank_o__Clostridia_UCG‐014, f__norank_o__Clostridia_UCG‐014, o__Clostridia_UCG‐014, g__norank_f__p‐251‐o5, f__p‐251‐o5, g__Lachnospira*, and *g__Coprococcus*. The most varied species in the CON were *g__unclassified_f__Micrococcaceae, o__Corynebacteriales, g__Corynebacterium, f__Corynebacteriaceae, g__Kocuria, o__Micrococcales, g__Roseburia*, and *g__Eubacterium_oxidoreducens showed* the most significant differences.

**TABLE 7:** Effects of lysolecithin on alpha diversity metrics of Holstein calves.

Item	Treatment^a^		SEM	*p*
	CON	LY		
ACE	1189.69	1300.21	75.88	0.49
Chao	1177.63	1287.73	73.33	0.48
Sobs	1170.00	1281.83	71.72	0.46
Shannon	5.91	6.20	< 0.10	0.14
Simpson	0.01	< 0.01	< 0.01	0.09
Coverage	1.00	1.00	< 0.01	0.95

^a^ CON, basal diet; LY, basal diet added 5 g/d lysolecithin.

### 3.6 Correlation Analysis of Rumen Microorganisms and Ruminal Fermentation

**FIGURE 2:**
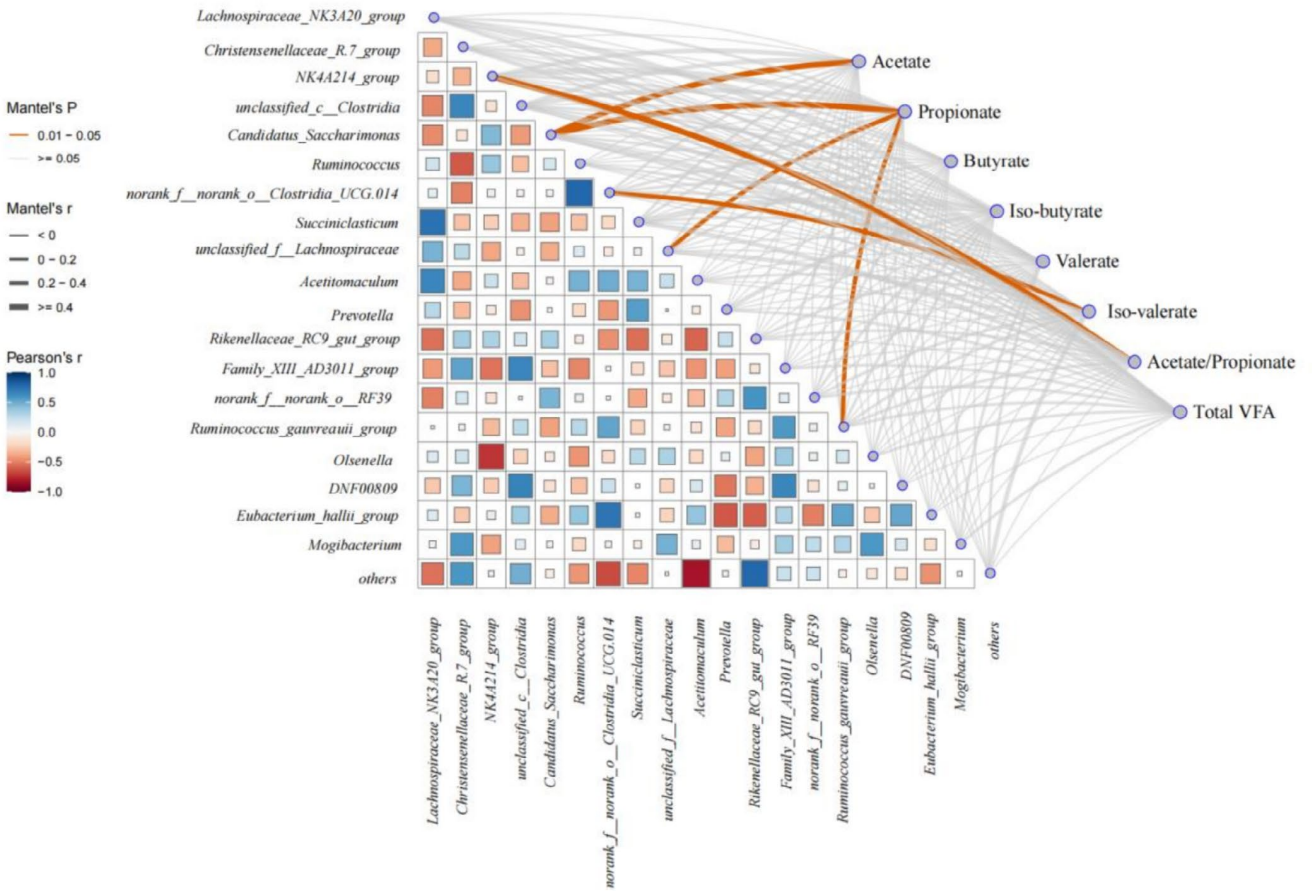
Correlation analysis between differentially bacterial genera and ruminal fermentation. The orange lines indicate significant correlations with Mantel’ test, and the thickness represents the strength and direction of the correlations.

The results of correlation between differentially abundant bacterial genera and ruminal fermentation showed that acetate was significantly positively correlated with *Candidatus_Saccharimonas* (Figure 2, *p* < 0.05). Propionate was positively correlated with *Ruminococcus_gauvreauii_group*, *Candidatus_Saccharimonas*, and *unclassified_f__Lachnospiraceae* (*p* < 0.05). Moreover, *NK4A214_group* was significantly positively correlated with acetate/propionate and the concentration of iso‐valerate was positively correlated with *norank_f__norank_o__Clostridia_UCG‐014* (*p* < 0.05).

## 4 Discussion

Calves exhibit rapid growth rates, whereas they also have high nutritional needs. Under weaning stress conditions, growth performance and health of Holstein calves can be affected (Enríquez et al. 2011). It is essential to enhance the ability to absorb and utilize nutrients effectively to meet the nutritional needs of calves. There were no significant differences in feed intake, weight, and body size when lysolecithin was added to the diet. Similarly, Khonyoung et al. (Khonyoung et al. 2015) found that feeding lysolecithin cannot improve growth performance for chickens.

The study found that the addition of lysolecithin to the diet did not impact fecal scores of dairy calves. Manure screen is used as a way of evaluating the nutrient utilization of diets (Kljak et al. 2019). This result shows that the feces of the bottom sieve in the LY were lower than in the CON, whereas the feces of the middle sieve in the LY were heavier than in the CON. It means that the rate of digestion and absorption was lower for the LY compared to the CON.

In this experiment, adding lysolecithin to the diet could increase serum ALB concentration, which may indicate a potential benefit for the immunity of dairy calves. The observation was that the concentration of BUN was reduced in the LY, indicating that rumen microbiota can effectively utilize ammonia and less ammonia is absorbed into the blood (Khezri et al. 2017). An imbalance between oxidative and antioxidative processes within calves can lead to oxidative stress (Surai et al. 2019). When dairy calves were fed lysolecithin, their serum T‐AOC increased. Similarly, the addition of emulsifier could enhance the antioxidant capacity of rainbow trout (Zhang et al. 2021). In this study, lysolecithin reduced the concentration of NEFA and TG, while increasing the concentration of TCH. NEFA plays a vital role in energy metabolism, whereas elevated levels can provoke inflammatory responses in cells, and TG can readily trigger inflammatory responses in dairy cows (Wagner et al. 2023). The previous study has shown that the supplementation of lysolecithin may reduce TG concentration by more effectively utilizing lipids (Upadhaya et al. 2018). Correspondingly, the TCH concentration increased during the process of promoting fat absorption and transport, which may lead to the lower TG concentration but higher TCH concentration of lysolecithin in this study. Nevertheless, the absence of a difference in GLU and the decrease in NEFA concentration in this study may be the reason why lysolecithin has no effect on the growth performance in Holstein calves (Agustinho et al. 2024). These results indicate that the supplementation of lysolecithin may reduce inflammatory responses and affect lipid metabolism in dairy calves.

NH_3_‐N is a degradation product in the rumen, serves as a significant nitrogen protein synthesis in large numbers of microorganisms (Hristov et al. 2004). In this study, the NH_3_‐N concentration in the rumen fluid was lower in the LY compared to the CON, which indicates NH_3_‐N is effectively utilized for microbial protein synthesis by rumen microbiota in the LY (Dewhurst and Newbold 2022). VFA is generated by the fermentation process carried out by rumen microorganisms, predominantly including acetate, propionate, and butyrate, which serve as a vital energy source for ruminants. In this study, there were no differences in the proportions of acetate and propionate between groups, suggesting that the addition of emulsifiers does not affect the fermentation type in the rumen of dairy calves. The addition of lysolecithin can significantly increase the proportions of butyrate, iso‐butyrate, valerate, and iso‐valerate in the rumen fluid of dairy calves. Butyrate can enhance the development of the ruminal epithelium and hence improve the digestive and absorptive capabilities (Mao et al. 2017).

Alpha diversity metrics were similar between groups, except the Simpson index tended to decrease with lysolecithin. This result indicates that ruminal bacterial diversity is not different. In this study, *Firmicutes*, *Bacteroidota*, and *Patescibacteria* were the core flora in the rumen, whereas no differences were observed. Results of LEfSe illustrated that *Firmicutes* predominated after lysolecithin supplementation, whereas *Actinobacteria* was the dominant microbial group in the CON. *Firmicutes* include the majority of cellulolytic bacteria, which can effectively decompose structural carbohydrates (de Melo et al. 2023). There were ruminal dominant bacteria including *Lachnospiraceae_NK3A20_group*, *Christensenellaceae_R‐7_group*, *NK4A214_group, unclassified_c__Clostridia, Candidatus_Saccharimonas*, and *Ruminococcus* at the genus level. In the current result, supplementation with lysolecithin increased the relative abundance of *Ruminococcus* but decreased *Corynebacterium*. *Ruminococcus* plays a crucial role in the degradation of cellulose and resistant starch in the rumen (Zhang et al. 2023). *Corynebacterium* is a pathogenic bacterium responsible for causing mastitis in dairy cattle. This means that lysolecithin may hold the potential to mitigate inflammatory responses (Panchal et al. 2024). Results of LEfSe illustrated that the relative abundances of *Ruminococcaceae* and *Lachnospiraceae* belonging to *Firmicutes* in the LY were more abundant than in the CON. Furthermore, they are linked to degrading dietary cellulose and greatly affect the digestion and nutrient absorption in ruminants (Lai et al. 2023). There is an interaction between rumen microbiota and VFA in the rumen. In this study, this is consistent with the previous finding that *Candidatus_Saccharimonas* can utilize carbohydrates to produce acetate and propionate (Pang et al. 2022). In addition, *norank_F_norank_O_Clostridia_UCG‐014* was positively correlated with iso‐valerate, which can promote the growth of cellulolytic microorganisms (Liu et al. 2021). This result showed that *NK4A214_group* was positively correlated with acetate/propionate. One possible explanation could be that *NK4A214_group* can degrade dietary fiber to produce butyrate and maintain the balance of the ruminal environment (Li et al. 2023). Propionate was positively correlated with *Candidatus_Saccharimonas*, *unclassified_f__Lachnospiraceae* and *Ruminococcus_gauvreauii_group*. This was similar to the study of Xiaoqian Lin et al. (2024), who reported that a large number of members of the *Lachnospiraceae* family have the ability to produce butyrate and propionate. *Candidatus_Saccharimonas* can promote amino acids, which provide nutrients for the rumen microbiota (Wang et al. 2023). *Ruminococcus_gauvreauii_group* can also degrade dietary fiber to produce VFA (Monteiro et al. 2022). Lysolecithin can enhance the growth of cellulose‐degrading bacteria in the rumen to produce corresponding VFA.

## 5 Conclusions

In conclusion, lysolecithin did not significantly affect the growth performance of Holstein calves, but can improve T‐AOC, affect plasma biochemical indices, increase percentage of some VFA, and reduce NH_3_‐Nconcentration.

## Author Contributions

**Zhigao An:** writing – original draft and reviewed the manuscript. **Shijia Pan:** investigation and writing – original draft. **Bo He:** investigation and data curation. **Junhong Wang:** investigation. **Yanming Wang:** investigation. **Shijie Sun:** investigation. **Chong Wang:** review, visualization, and funding acquisition. All authors read and approved the final manuscript.

## Funding

This work was supported by the National Natural Science Foundation of China (32172742), the Zhejiang Provincial Science and Technology Plan Project (2025C04036), the Cooperative Extension Plan of Major Agricultural Technologies of Zhejiang Province (2025ZDXT15‐03), the Yinchuan Science and Technology Plan Project (2023XTCX02) and the Jiaxing Science and Technology Plan Project (2025CGZ073, 202401017).

## Conflicts of Interest

The authors declare no conflicts of interest.

## Data Availability

The data that support the findings of this study are available from the corresponding author upon reasonable request.
